# Cholinergic epithelial cell with chemosensory traits in murine thymic medulla

**DOI:** 10.1007/s00441-014-2002-x

**Published:** 2014-10-10

**Authors:** Alexandra Regina Panneck, Amir Rafiq, Burkhard Schütz, Aichurek Soultanova, Klaus Deckmann, Vladimir Chubanov, Thomas Gudermann, Eberhard Weihe, Gabriela Krasteva-Christ, Veronika Grau, Adriana del Rey, Wolfgang Kummer

**Affiliations:** 1 Institute of Anatomy and Cell Biology, Justus-Liebig-University Giessen, Aulweg 123, 35385 Giessen, Germany; 2 Institute of Anatomy and Cell Biology, Philipps-University Marburg, Marburg, Germany; 3 Walter-Straub-Institute for Pharmacology and Toxicology, Ludwig-Maximilian-University, Munich, Germany; 4 German Center for Lung Research, Giessen, Germany; 5 Department of General and Thoracic Surgery, Laboratory of Experimental Surgery, Justus-Liebig-University Giessen, Giessen, Germany; 6 Department of Immunophysiology, Institute of Physiology and Pathophysiology, Medical Faculty, Philipps-University Marburg, Marburg, Germany

**Keywords:** Acetylcholine, Brush cell, Chemosensory, Taste transduction, Thymus, Mouse

## Abstract

Specialized epithelial cells with a tuft of apical microvilli (“brush cells”) sense luminal content and initiate protective reflexes in response to potentially harmful substances. They utilize the canonical taste transduction cascade to detect “bitter” substances such as bacterial quorum-sensing molecules. In the respiratory tract, most of these cells are cholinergic and are approached by cholinoceptive sensory nerve fibers. Utilizing two different reporter mouse strains for the expression of choline acetyltransferase (ChAT), we observed intense labeling of a subset of thymic medullary cells. ChAT expression was confirmed by in situ hybridization. These cells showed expression of villin, a brush cell marker protein, and ultrastructurally exhibited lateral microvilli. They did not express neuroendocrine (chromogranin A, PGP9.5) or thymocyte (CD3) markers but rather thymic epithelial (CK8, CK18) markers and were immunoreactive for components of the taste transduction cascade such as Gα-gustducin, transient receptor potential melastatin-like subtype 5 channel (TRPM5), and phospholipase C_β2_. Reverse transcription and polymerase chain reaction confirmed the expression of Gα-gustducin, TRPM5, and phospholipase C_β2_. Thymic “cholinergic chemosensory cells” were often in direct contact with medullary epithelial cells expressing the nicotinic acetylcholine receptor subunit α3. These cells have recently been identified as terminally differentiated epithelial cells (Hassall’s corpuscle-like structures in mice). Contacts with nerve fibers (identified by PGP9.5 and CGRP antibodies), however, were not observed. Our data identify, in the thymus, a previously unrecognized presumptive chemosensitive cell that probably utilizes acetylcholine for paracrine signaling. This cell might participate in intrathymic infection-sensing mechanisms.

## Introduction

The thymus is the site of maturation of naive T cells from immature thymocytes, which are derived from progenitors recruited from the bone marrow. During maturation, thymocytes migrate from the cortex to the medulla and differentiate from a CD4^-^CD8^-^ (double-negative) to a CD4^+^CD8^+^ (double-positive) and finally to a CD4^+^ or CD8^+^ single-positive phenotype. Along the way, thymocytes are subjected to positive and negative selection processes in the cortex and medulla, respectively, for which specialized subsets of thymic epithelial cells are indispensable. From the medulla, naive mature thymocytes are released into the circulation and are recruited to secondary lymphatic tissues. These processes are influenced by cholinergic signaling mechanisms, as low-dose nicotine arrests thymocyte maturation at the double-positive stage in fetal murine thymus organ culture (Middlebrook et al. 2002). Moreover, evidence exists that cholinergic agonists trigger the release of mature lymphocytes into the circulation (Maśliński et al. 1987; Antonica et al. 1994). Acetylcholine (ACh) is endogenously synthesized in the thymus (Rinner et al. 1999; Fujimoto et al. 2001), and expression of the ACh-synthesizing enzyme choline acetyltransferase (ChAT) is strongly upregulated in the murine thymus during the first two months after birth (Tria et al. 1992). Whereas the extent and relevance of the cholinergic innervation of the thymus have been controversially discussed (Fatani et al. 1986; Singh et al. 1987; Dorko et al. 2011), ACh synthesis has also been ascribed to subsets of thymic epithelial cells and lymphocytes (Tria et al. 1992; Rinner et al. 1999; Kawashima and Fujii 2004), although the definitive in situ identification of ACh producing cells in the thymus has not been unequivocally achieved as yet.

We have here utilized two independently generated BAC transgenic mouse strains expressing enhanced green fluorescent protein (eGFP) driven by the ChAT promoter (Tallini et al. 2006; Engelhardt et al. 2007) in order to identify intrinsic cholinergic cells in the murine thymus. ChAT-eGFP expression has been noted in thymic epithelial cells that, in shape, resemble solitary chemosensory cells recently identified in the mucosa of the respiratory tract and stomach (Ogura et al. 2010; Krasteva et al. 2011; 2012; Eberle et al. 2013). Such cells are characterized by an apical tuft of villin-containing microvilli (hence also termed “brush cells”) and by the expression of the signal transduction cascade for canonical bitter and sweet/umami perception, i.e., the Gα-protein Gα-gustducin, phospholipase C_β2_ (PLCβ2), and transient receptor potential melastatin-like subtype 5 channel (TRPM5; Krasteva and Kummer 2012; Eberle et al. 2013), a voltage-modulated and Ca^2+^-activated monovalent selective cation channel (Hofmann et al. 2003). They utilize the canonical taste transduction cascade to detect bacterial substances and initiate aversive protective reflexes utilizing cholinergic signaling to sensory neurons (Ogura et al. 2010; Krasteva et al. 2011; Saunders et al. 2014). This has led to the concept that taste receptors have the quality of a novel class of pathogen receptor, and that these cholinergic chemosensory cells serve as sentinels at the portals of entry into organ systems lined by a mucosal surface (Fatani et al. 1986). In view of their structural similarity to the intrinsic cholinergic cells in the murine thymus, our present aim has been to determine whether these thymic cholinergic cells also express components of the canonical taste transduction cascade and villin, a molecular marker of solitary chemosensory cells.

## Materials and methods

### Mice

ChAT^BAC^-eGFP transgenic mice (Tallini et al. 2006; Engelhardt et al. 2007) of both genders, aged 6 days to 5 months, served as a tool for the study of the occurrence of cholinergic cells in the mouse thymus by (immuno-)fluorescence microscopy (*n* = 14) and in situ hybridization (*n* = 4). Samples for electron microscopy (*n* = 4) and for analysis by reverse transcription and polmerase chain reaction (RT-PCR; *n* = 3) were obtained from wild-type mice (C57Bl/6N). Transgenic mice expressing eGFP under the *chrna3* promoter (Frahm et al. 2011) (*n* = 5, aged 6 to 14 weeks, both genders) served as a tool to visualize the expression of the α3 nicotinic ACh receptor (nAChR) subunit. The housing, breeding, and usage of mice employed in this study were approved by the appropriate regional authorities (Rp Giessen, Germany; ref. numbers A9/2011, A60/2012, A61/2012).

### Immunohistochemistry

Mice were killed by the inhalation of an overdose of isoflurane (Abbott, Wiesbaden, Germany) and were either dissected freshly (*n* = 6) or were transcardially perfused with rinsing solution (Forssmann et al. 1977) followed by Zamboni fixative (2 % paraformaldehyde [PFA] in 0.1 M phosphate buffer and 15 % saturated picric acid, pH 7.4; *n* = 7) or 4 % phosphate-buffered PFA (*n* = 1 for ChAT^BAC^-eGFP mice and *n* = 5 for Chrna3^BAC^-eGFP mice). Thymi and organs serving as positive controls (adrenal gland, gut, spinal cord, tongue, and trachea) were dissected and either fixed by overnight immersion in Zamboni fixative (*n* = 3) or in 4 % PFA (*n* = 3) or, in the case of perfusion-fixed specimens, in the same fixative for another 5–6 h. Specimens were then repeatedly washed in buffer, rinsed overnight in 18 % sucrose in 0.1 M phosphate buffer, pH 7.4, and then frozen in OCT compound (Sakura Finetek, Staufen, Germany) by using isopentane cooled with liquid nitrogen. Specimens were stored at −80 °C until further use.

Cryosections (10 μm) were air-dried for 1 h, and unspecific protein-binding sites were saturated with blocking solution containing 10 % normal horse serum, 0.5 % Tween 20, and 0.1 % bovine serum albumin in PBS (0.005 M phosphate buffer, pH = 7.4, with 0.45 % NaCl) for 1 h. The sections were incubated with primary antibodies (Table 1) diluted in 0.005 M phosphate buffer containing 0.01 % NaN_3_ and 0.9 % NaCl overnight at room temperature in a dark and humid chamber. Next, the sections were rinsed with PBS, incubated with fluorophore-conjugated secondary antibodies (Cy3-conjugated anti-rabbit-Ig, Chemicon, Temecula, Calif., USA, 1:2000; Cy3-conjugated anti-goat-Ig, Chemicon, 1:800; fluorescein-isothiocyanate-conjugated anti-chicken-IgY, Dianova, Hamburg, Germany, 1:800; all raised in donkey) for 1 h, washed, and fixed for another 10 min in 4 % PFA. Rinsed sections were coverslipped with Mowiol 4–88 (pH 8.6; Merck, Darmstadt, Germany) containing 0.1 % 1,4-diazabicyclo[2.2.2]octane (Sigma, St. Louis, USA) or with carbonate-buffered glycerol (pH 8.6). The slides were evaluated with an epifluorescence microscope (Axioplan 2, Zeiss, Jena, Germany) or, in the case of labeling with anti-CD3, by confocal laser scanning microscopy (Olympus BX50WI, Olympus, Hamburg, Germany). Image processing (PowerPoint, Adobe Photoshop) was restricted to overall adjustment of brightness and contrast.

**Table 1:** Primary antibodies used in immunohistochemistry (*m* monoclonal, *p* polyclonal, *CGRP* calcitonin gene-related peptide, *CGA* chromogranin A, *CK* cytokeratin, *eGFP* enhanced green fluorescent protein, *PGP9.5* protein gene product 9.5, *PLCβ2* phospholipase C_β2_, *TRPM5* transient receptor potential melastatin-like subtype 5 channel)

Antigen	Host species	Clone/code	Working dilution	Source
CGRP	Goat, p	BT17-2090-07	1:4000	Biotrend, Cologne, Germany
CD3	Rabbit, p	A452	1:600	DAKO, Hamburg, Germany
CGA	Rabbit, p	1782-1	1:400	Epitomics, Burlingame, Calif., USA
CK5	Rabbit, m	SP27/M3270	1:200	Spring Bioscience, Pleasanton, Calif., USA
CK14	Rabbit, m	SP53/M3530	1:400	
CK8	Rabbit, m	SP102/M4020	1:100	
CK18	Rabbit, m	SP69/M3690	1:200	
eGFP	Chicken, p	NB 100-1614	1:16,000	Novus Biologicals, Littleton, Colo., USA
Gα-gustducin	Rabbit, p	sc-395	1:3000	Santa Cruz Biotechnology, Heidelberg, Germany
PGP9.5	Rabbit, p	BT78-6305-04	1:8000	Biotrend
PLCβ2	Rabbit, p	sc-206	1:800	Santa Cruz Biotechnology
TRPM5	Rabbit, p	ø	1:4000	Kaske et al. (2007)
Human villin	Rabbit, p	V2121-95	1:50	US Biological, Salem, Mass., USA
Chicken villin	Rabbit, p	ø	1:6400	Drenckhahn et al. (1983)

Specificity controls included preabsorption of the primary antibody with cognate peptide (sc-395 P for anti-Gα-gustducin, sc-206 P for anti-PLCβ2; both from Santa Cruz Biotechnology, Heidelberg, Germany) for 6 h at room temperature at a concentration of 20 μg peptide per 100 μl primary antibody at working dilution, replacement of primary antibody by normal rabbit serum, and omission of primary antisera.

### Pre-embedding immunohistochemistry and electron microscopy

Thymi from C57Bl/6N mice fixed by transcardiac perfusion with 4 % PFA were cryosectioned (40 μm), and free-floating sections were processed as described in detail earlier (Krasteva et al. 2011). Briefly, tissue sections were incubated overnight with rabbit anti-PLCβ2 (1:800, Santa Cruz Biotechnology), rabbit anti-TRPM5 (1:4,000, Kaske et al. 2007), or rabbit anti-human villin (1:50, US Biological, Salem, Mass., USA), and immunoreactivity was visualized with a peroxidase-based technique with peroxidase-conjugated porcine anti-rabbit Ig (1:100, Dako, Hamburg, Germany) serving as the secondary antibody and by utilizing nickel-ammonium-sulphate-enhanced diaminobenzidine as the chromogen. Cryosections were then osmicated, stained with uranyl acetate *en bloc*, routinely embedded for electron microscopy, trimmed for regions containing labeled cells, and sectioned for electron microscopy. Thin sections were stained with uranyl acetate and evaluated with an EM 902 transmission electron microscope (Zeiss, Jena, Germany).

### In situ hybridization

The thymus was quickly dissected and directly frozen in −40 °C cold isopentane. Serial 14-μm-thick sections were cut with a cryostat and mounted on silanized glass slides. Complementary RNA probes for the detection of mouse ChAT transcripts in tissue sections were generated from mouse C57BL/6 brainstem cDNA. First, a 758-nucleotide DNA fragment (GeneBank accession no. NM_003891, nucleotides 1468–2225) was amplified by PCR and subcloned into pGEM-T (Promega, Mannheim, Germany), and the sequence was confirmed by double-stranded sequencing. Then, radioactively (35S)-labeled antisense and sense riboprobes were generated by using SP6 (for the antisense probe) and T7 (for the sense probe) RNA polymerases. The in situ hybridization procedure was essentially performed as described in detail earlier (Schäfer et al. 1997; Schütz et al. 2000).

### Analysis by RT-PCR

RT-PCR analysis was conducted both on RNA extracted from whole thymus and from isolated presumptive (TRPM5-positive) cells by using a modification of a protocol described previously (Deckmann et al. 2014). Briefly, thymi were digested by means of collagenase II solution (1 mg/ml; Biochrom, Berlin, Germany) in Hank's balanced salt solution (HBSS; Invitrogen, Darmstadt, Germany) at 37 °C for 40 min. The cell suspension was filtered through a cell strainer (70 μm; BD Falcon, Heidelberg, Germany), washed with HBSS, and centrifuged at 1500 rpm for 5 min. This cell mixture was incubated with a rabbit polyclonal TRPM5 antibody directed against an extracellular epitope (1:125; ab72151, Abcam, Cambridge, England) for 1 h at 37 °C, followed by purification by using magnetic beads coated with goat anti-rabbit IgG (PI65-6100, Invitrogen).

From both isolated cells and whole thymi, RNA was isolated with RNeasy mini-kit (Qiagen, Hilden, Germany) according to the manufacturer’s instructions. Genomic DNA was eliminated by treating 1 μg total RNA with 1 U DNAse (Invitrogen) for 15 min at 25 °C with a subsequent switch to 65 °C for 10 min in the presence of 1 μl of 25 mM EDTA (Invitrogen). cDNA was synthesized with 1 μl oligo-dT primers (0.5 μg/μl; MWG Eurofins, Munich, Germany) and 1 μl dNTPs (10 mM each; Invitrogen) in the presence of 2 μl dithiothreitol (0.1 M), 200 U Superscript II reverse transcriptase, and 4 μl 5xRT buffer (Superscript II reverse transcriptase kit, Invitrogen) at 42 °C for 50 min, with a subsequent switch to 70 °C for 10 min. PCR was performed by mixing 2.5 μl cDNA, 2.5 μl MgCl_2_, 2.5 μl Buffer II (100 mM TRIS–HCl, 500 mM KCl, pH 8.3), 0.25 μl AmpliTaq DNA polymerase (5 U/μl; all three from Applied Biosystems, Darmstadt, Germany), 0.75 μl dNTPs (10 mM each), 0.75 μl primer mix (sequences are indicated in Table 2; Eurofins MWG) and water up to 25 μl. PCR conditions were 10 min at 95 °C, 40 cycles of 20 s at 95 °C, 20 s at 60 °C, and 20 s at 72 °C. β-Actin was used as a control for PCR, whereas omission of the reverse transcriptase during cDNA synthesis served as a control for DNAse digestion efficiency and reagent cleanliness. The PCR products were separated and visualized on ethidium-bromide-supplemented 2 % TRIS-acetate-EDTA agarose gel.

**Table 2:** Primers used for reverse transcription with the polmyerase chain reaction (*fw* forward, *rev* reverse)

Target	Sequence (5′-3′)	Product length	GenBank accession umber.
β-Actin	fw: gtgggaatgggtcagaagg rev: ggcatacagggacagcaca	300 bp	NM_007393.3
Gα-gustducin	fwd: tcatccataagaatggttacagc rev: cccacagtcgtttaatgatttc	231 bp	NM_001081143
PLCβ2	fw: ttccagatgtttcctgctga rev: gggaagtcctctgggttgat	101 bp	NM_177568
TRPM5	fw: tgaggaacgacctttggcta rev: acacggatcttggtggatgt	183 bp	NM_020277.2

## Results

### A thymic medullary epithelial cell is cholinergic

**Fig. 1:**
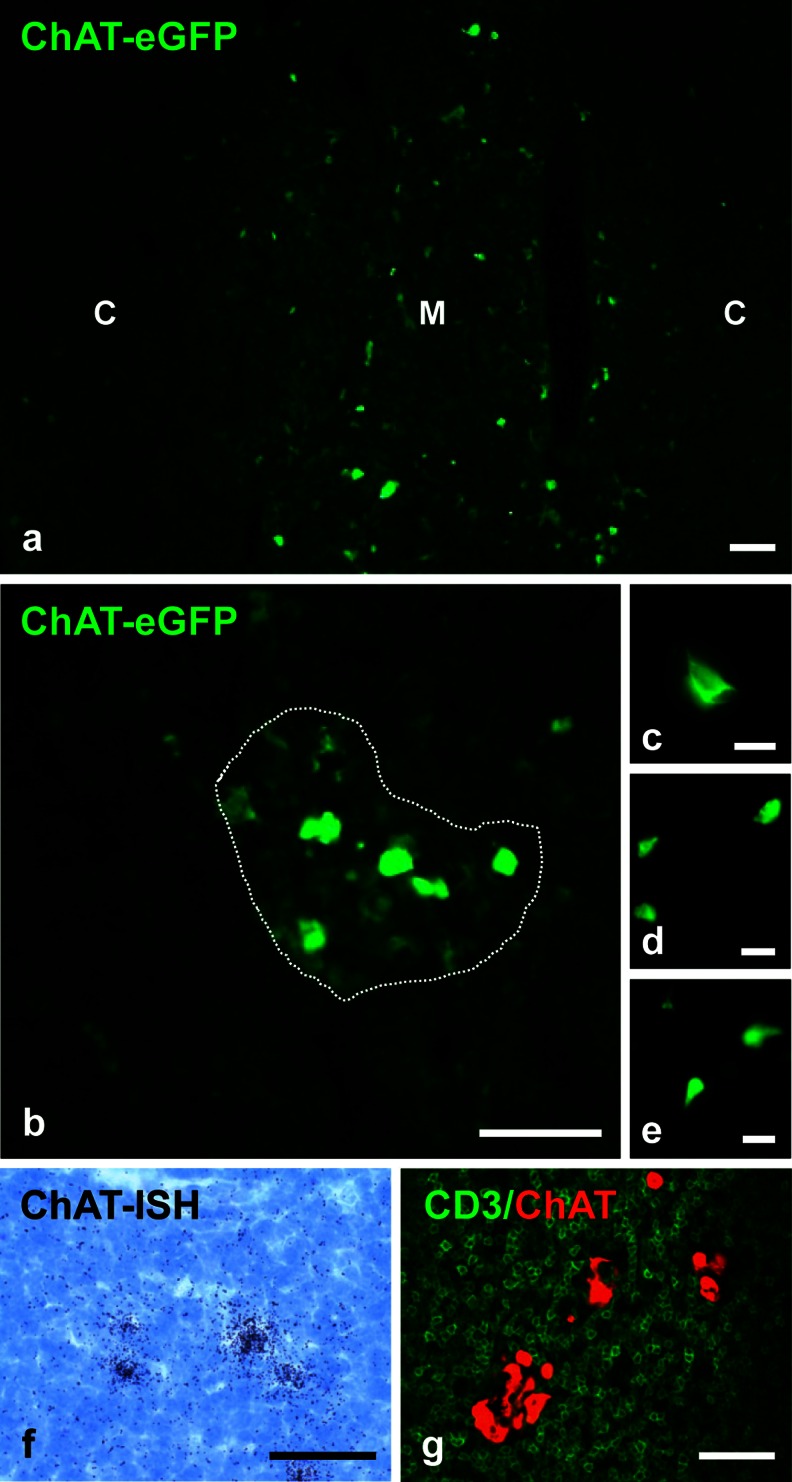
Cholinergic cells reside in the thymic medulla. **a–e** Choline acetyltransferase (*ChAT*)-enhanced green fluorescent protein (*eGFP*) fluorescence (*C* cortex, *M* medulla). Positive cells are scattered throughout the medulla (**a**), and some form loose clusters (*dotted line* in **b**). **c–e** Higher magnification reveals oval- to triangular-shaped cells with short cellular extensions. **f** Radioactive in situ hybridization (*ISH*) for ChAT-mRNA demonstrating labeled cells in the thymic medulla of a wild-type mouse. **g** ChAT-eGFP fluorescence enhanced by eGFP immunolabeling (*red*), CD3 immunolabeling (*green*); confocal laser scanning microscopy. ChAT-eGFP does not colocalize with CD3. **a**, **b** Male aged 25 weeks. **c** Female aged 25 weeks. **d** Male aged 31 weeks. **e** Male aged 22 weeks. **f** Male aged 17 weeks. **g** Male aged 19 weeks. *Bars* 50 μm (**a**, **b**, **f**, **g**), 10 μm (**c**), 20 μm (**d**, **e**)

In both mouse strains, intense ChAT^Bac^-eGFP fluorescence was observed in scattered cells in the thymic medulla, partly forming loose clusters, with a preference for the outer medulla (Fig. 1a, b). We did not note distinct changes in this arrangement over time in the age range investigated. Cellular section profiles ranged from round to triangular, elongated or pear-shaped with one, occasionally two, cellular extensions (Fig. 1b). In situ hybridization confirmed ChAT-mRNA expression in singly lying cells in the medulla (Fig. 1c). No labeled cells were observed in thymic sections exposed to sense riboprobes.

When eGFP fluorescence was enhanced by anti-eGFP immunolabeling by utilizing fluorophore- or peroxidase-conjugated secondary antibodies, additional, less intensely stained cells of dendritic morphology forming a medullary network were visible in some but not all preparations. The further characterization of ChAT-eGFP cells will not refer to these weakly stained cells.

**Fig. 2:**
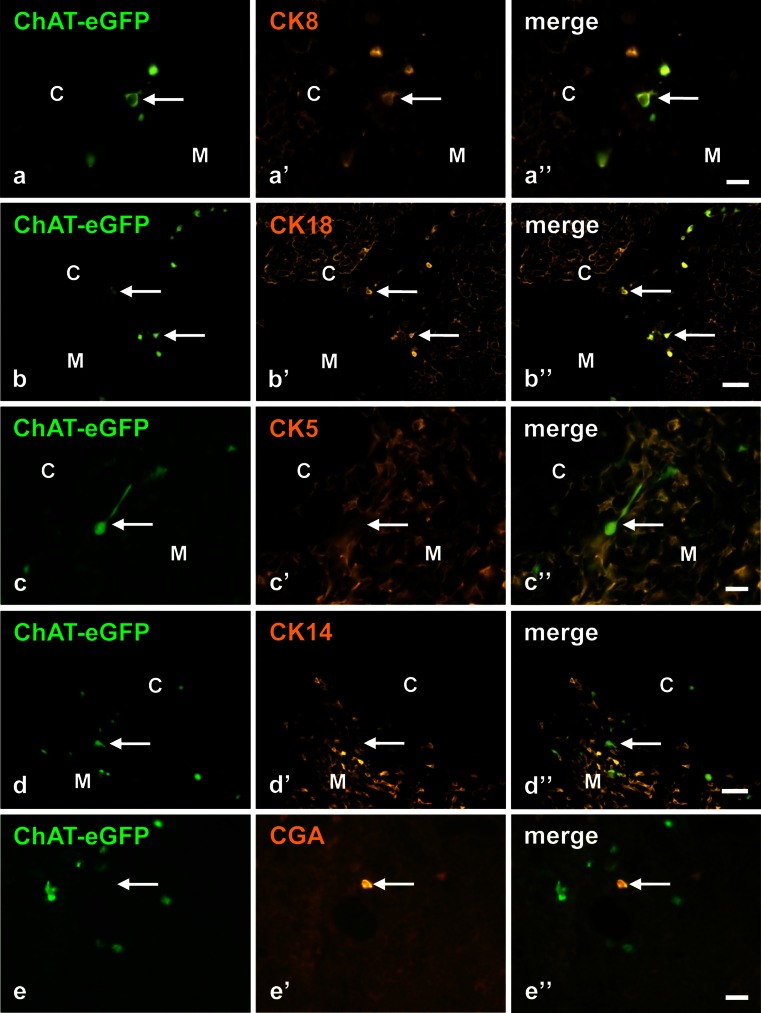
ChAT-eGFP-positive cells are thymic epithelial cells (*C* cortex, *M* medulla). ChAT-eGFP-positive cells are immunoreactive for CK8 (**a–a”**) and CK18 (**b–b”**) but are immunoreactive for neither CK5 (**c–c”**) nor CK14 (**d–d”**). ChAT-eGFP-positive cells do not contain CGA immunoreactivity (**e–e”**). **a–d** Female aged 25 weeks. **e** Male aged 25 weeks. *Bars* 20 μm (**a**, **c**, **e**), 50 μm (**b**, **d**)

Immunohistochemistry for CD3, a T-cell co-receptor expressed in thymocytes and all mature T-cells, revealed that the cholinergic cells were not lymphocytes (Fig. 1g). Cytokeratins (CK) are markers for thymic epithelial cells, with a preferential expression of CK8 and CK18 by cortical epithelial cells and CK5/CK14 by medullary epithelial cells (Shezen et al. 1995; Klug et al. 1998; Liepinsh et al. 2009; Lee et al. 2011). We observed immunolabeling for CK8 and CK18 in eGFP^+^ cells in the medulla next to the cortico-medullary border (Fig. 2a, b), even though CK8 and CK18 are generally considered as being typical for cortical epithelial cells. Conversely, although being located in the medulla, ChAT-eGFP cells did not contain typical medullary cytokeratins, i.e., CK5 and CK14 (Fig. 2c, d). Chromogranin A (CGA) has been reported in neuroendocrine cells of the thymus, including carcinoid tumors derived from them, and in a subset of terminally differentiated medullary epithelial cells (Herbst et al 1987; Brelińska et al. 2000; Soultanova et al. 2014). The cholinergic cells in the mouse thymus were distinct from these cells, since CGA did not co-localize with ChAT-eGFP (Fig. 2e).

### Cholinergic medullary epithelial cells express villin and the canonical taste transduction cascade

**Fig. 3:**
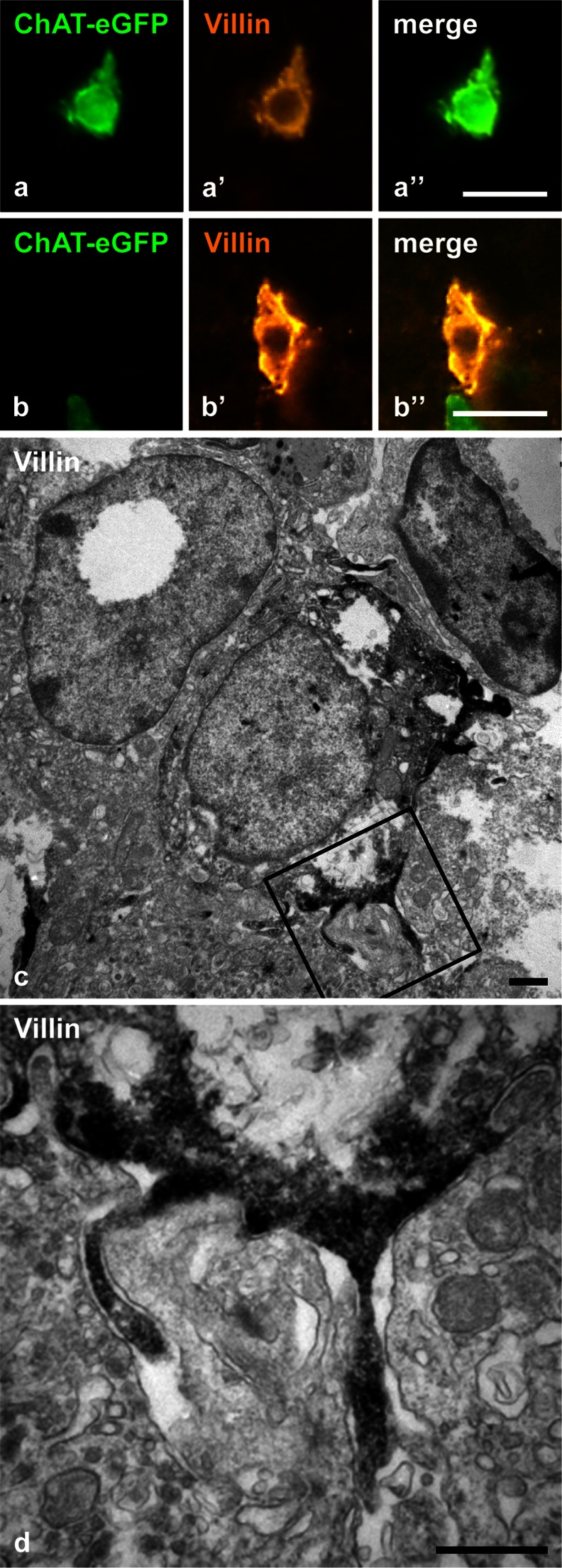
Villin immunolabeling. ChAT-eGFP-positive cell with immunoreactivity for villin (**a–a”**) and villin-positive cell lacking ChAT-eGFP-fluorescence (**b–b”**). **c**, **d** Ultrastructural immunohistochemistry, pre-embedding technique. Villin immunoreactivity is indicated by *dark* diaminobenzidine reaction product. A villin-immunoreactive epithelial cell extends lateral microvilli. **d** Higher magnification of *boxed area* in **c**. **a** Male aged 31 weeks. **b** Female aged 25 weeks. **c**, **d** Female aged 9 weeks. *Bars* 20 μm (**a**, **b**), 1 μm (**c**), 0.5 μm (**d**)

Double-labeling revealed extensive co-localization of villin immunoreactivity with ChAT-eGFP expression in medullary epithelial cells (Fig. 3a), although some single-positive cells (villin^+^/eGFP^-^ and villin^-^/eGFP^+^) were also noted (Fig. 3b). At the ultrastructural level, villin-immunoreactive cells exhibited lateral microvilli extending between neighboring epithelial cells (Fig. 3c, d), as has been previously described for brush cells in the gut (Höfer and Drenckhahn 1992).

**Fig. 4:**
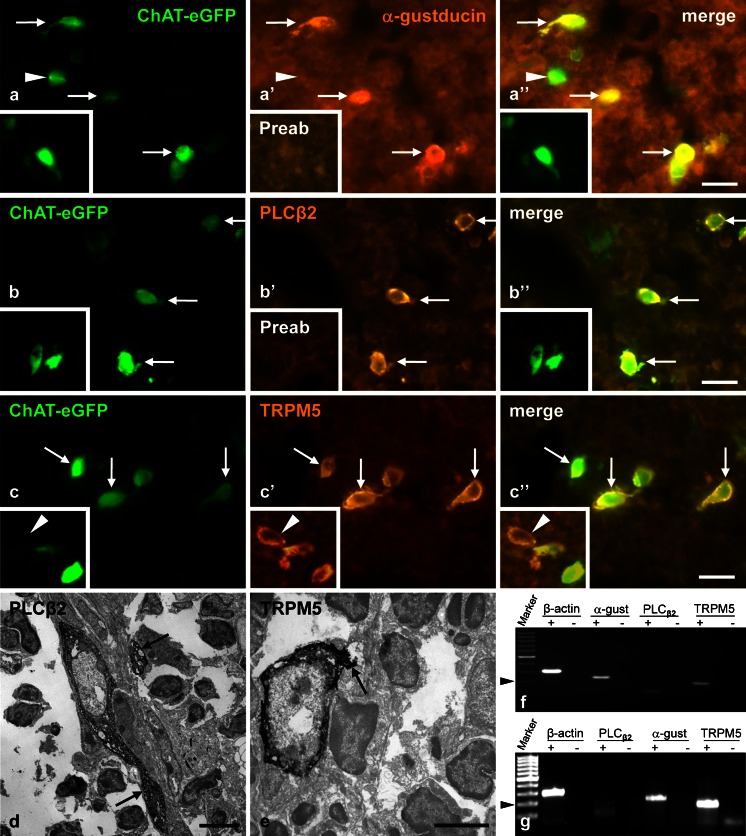
Cholinergic medullary epithelial cells express components of the canonical taste transduction cascade. **a–c** Most ChAT-eGFP-positive epithelial medullary cells are immunoreactive (*arrows*) for the downstream signaling components of the canonical bitter, sweet, and umami taste transduction cascade, namely Gα-gustducin (*α-gustducin*), phospholipase C_β2_ (*PLCβ2*), and transient receptor potential melastatin-like subtype 5 channel (*TRPM5*). *Insets* in **a** and **b** Preabsorption controls (*Preab*) in which the primary antibody had been saturated with cognate peptide prior to application to the tissue section. *Arrowheads* in **a** and in *inset* in **c** indicate single cells displaying only one marker. *Bars* 20 μm. **d**, **e** Ultrastructural immunohistochemistry, pre-embedding technique. PLCβ2-immunoreactive (**d**) and TRPM5-immunoreactive (**e**) epithelial cells extending processes (*arrows*). **a**, **b** Male aged 25 weeks. *Inset* in **a**, **b** Male aged 16 weeks. **c** Male aged 31 weeks. *Inset* Female aged 25 weeks. **d**, **e** 31 weeks. *Bars* 5 μm. **f**, **g** RT-PCR, ethidium-bromide-stained agarose gels. Gα-gustducin-specific (*α-gust*), PLCβ2-specific (weakly), and TRPM5-specific products were amplified from whole thymus (**f**) and thymic cells isolated by aid of TRPM5-antibody and magnetic beads (**g**). *Arrowhead* indicates 200 bp marker. PCR was conducted with (*+*) and without (*-*; serving as negative control) reverse transcription of RNA. β-Actin served as a housekeeping gene

The majority of ChAT-eGFP-positive epithelial medullary cells were immunoreactive for the downstream signaling components of the canonical bitter, sweet, and umami taste transduction cascade, i.e., Gα-gustducin, PLCβ2, and TRPM5, although eGFP-positive cells without additional immunoreactivities were also observed (Fig. 4a–c). Few cells with immunoreactivity for PLCβ2 or TRPM5 that did not express ChAT-eGFP were present, whereas Gα-gustducin-immunoreactive cells lacking ChAT-eGFP were practically absent. Ultrastructural immunohistochemistry revealed that medullary PLCβ2- and TRPM5-immunoreactive cells were epithelial cells extending long processes (Fig. 4d, e).

RT-PCR confirmed the expression of mRNA coding for Gα-gustducin, PLCβ2, and TRPM5 in whole thymus and in isolated presumptive chemosensory cells (Fig. 4f, g).

### Presumptive medullary chemosensory cholinergic cells are in contact with cholinoceptive epithelial cells rather than being innervated

**Fig. 5:**
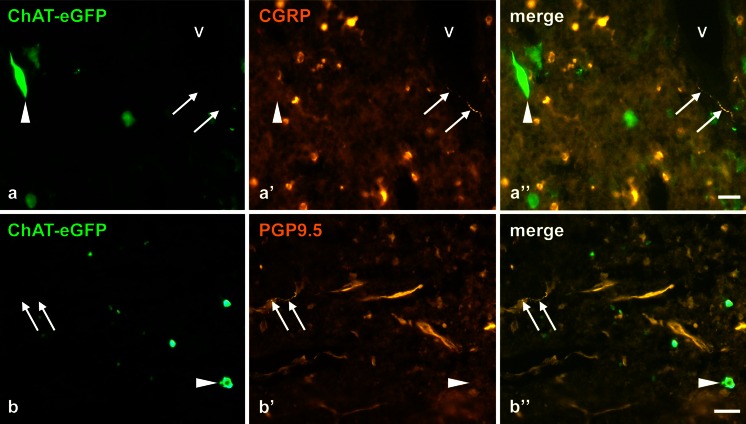
Cholinergic medullary epithelial cells are not innervated. **a** Calcitonin gene-related peptide (*CGRP*)-immunoreactive varicose nerve fibre (*arrows*) next to a medullary blood vessel (*V*). However, the ChAT-eGFP-positive cell with an elongated process (*arrowhead*) is not approached by a nerve fibre. **b** Non-innervated ChAT-eGFP-positive cell (*arrowhead*) distant from a medullary protein gene product 9.5 (*PGP9.5*)-immunoreactive varicose nerve fibre (*arrows*). **a** Female aged 25 weeks. **b** Female aged 17 weeks. *Bars* 20 μm (**a**), 50 μm (**b**)

Chemosensory cells in respiratory epithelia are approached by peptidergic (substance P, calcitonin gene-related peptide [CGRP]) sensory nerve fibers (Finger et al. 2003; Krasteva et al. 2011; Saunders et al. 2014). In agreement with the observations reported previously by Bulloch et al. (1991), immunolabeling for CGRP revealed small CGRP-immunoreactive cortico-medullary cells and nerve terminals in septa and along blood vessels (Fig. 5a). These fibers did not ramify within the medulla nor did they approach ChAT-eGFP-positive cells (Fig. 5a). To test for possible innervation by non-peptidergic sensory nerve fibers, antibodies directed against the general neuroendocrine marker, protein gene product 9.5 (PGP9.5), were used. This antibody also labeled nerve fibers in septa and around blood vessels, but not in the vicinity of ChAT-eGFP-positive cells (Fig. 5b). As reported earlier by other groups (Brelińska et al. 2000; Bai et al. 2008), PGP9.5 immunoreactivity in the thymus was not restricted to nerve fibers but was also observed in several other cell types. ChAT-eGFP-positive cells represented a cell population distinct from these PGP9.5-immunoreactive thymic cells (Fig. 5b).

**Fig. 6:**
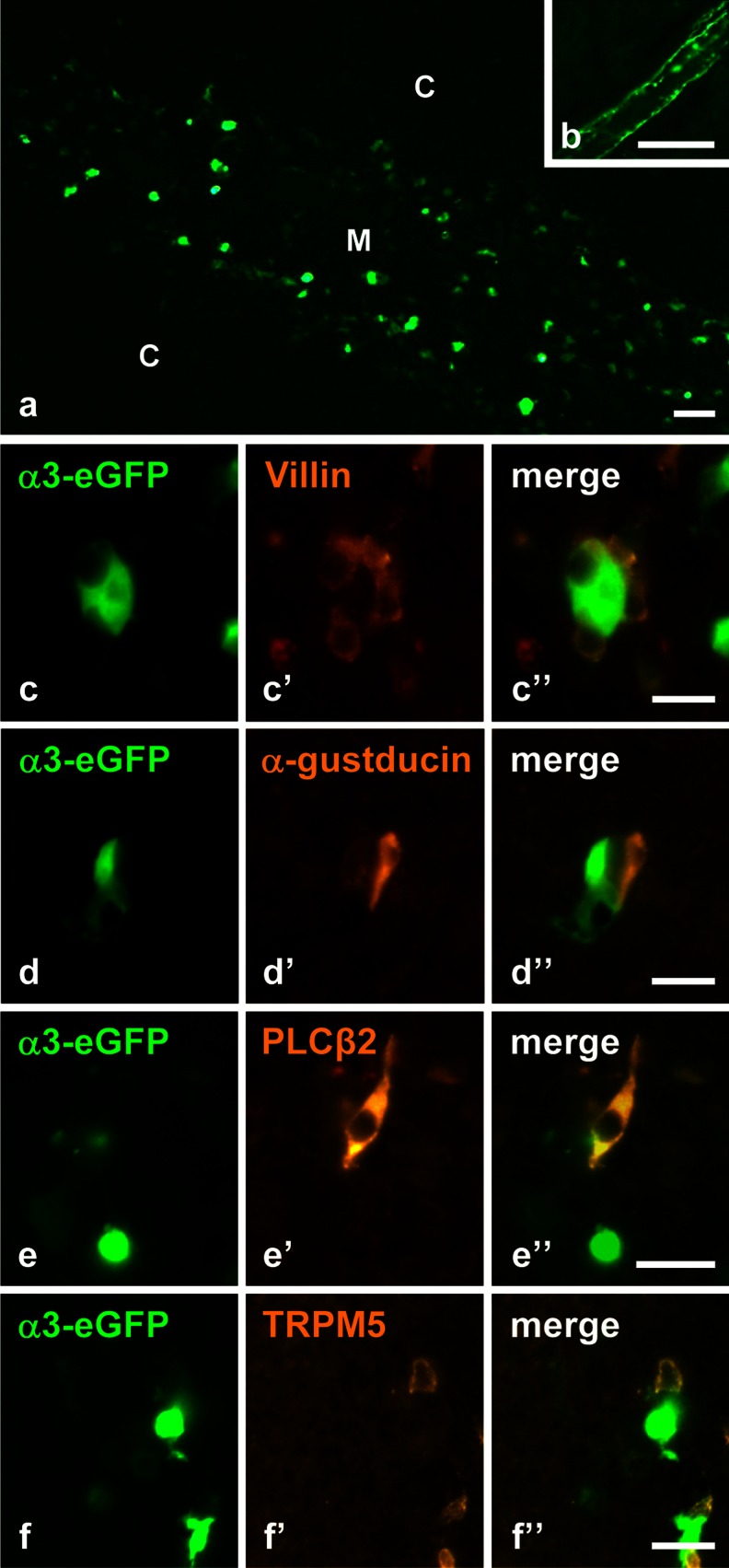
Cells expressing the nicotinic ACh receptor (*nAChR*) α3-subunit often are in close proximity to presumptive chemoreceptive cells. **a**, **b** nAChRα3-eGFP-fluorescence. **a** Positive cells are scattered throughout the medulla. **b** Nerve fibers enmesh a small septal artery. **c–f** nAChRα3-eGFP-positive cells lying next to cells immunoreactive for villin (**c**), α-gustducin (**d**), PLCβ2 (**e**), and TRPM5 (**f**). **a**, **b** Male aged 6 weeks. **c**, **d** Male aged 14 weeks. **e**, **f** Female aged 8 weeks. *Bars* 50 μm (**a**, **b**), 20 μm (**c–f**)

In the respiratory and urethral epithelium, sensory nerve fibers expressing the nAChRα3-subunit establish direct contacts with chemosensory cells (Krasteva et al. 2011; Deckmann et al. 2014). Utilizing the same Chrna3^BAC^-eGFP mouse strain as in these previous studies, we noted positive perivascular axons and nerve fibers in the septa, but not in the thymic medulla unrelated to vessels. As also reported recently (Soultanova et al. 2014), however, nAChRα3-subunit-positive epithelial medullary cells were seen (Fig. 6a, b). Co-immunolabeling with antibodies directed against villin and the components of the taste transduction cascade (Gα-gustducin, PLCβ2, and TRPM5) revealed that presumptive chemosensory cells and nAChRα3-subunit-expressing cells often formed small clusters and were in immediate contact with each other but always represented distinct cell populations (Fig. 6c–f).

## Discussion

The present study describes a hitherto unrecognized epithelial cell phenotype that resides in the medulla of the mouse thymus and that shares several hallmark features with chemosensory cells of the airway mucosa: 1) villin-containing microvilli, 2) expression of the downstream components of the canonical bitter and sweet/umami taste transduction cascade, i.e., Gα-gustducin, PLCβ2, and TRPM5, and 3) ChAT, the acetylcholine-synthesizing enzyme. Provisionally, we propose the term thymic cholinergic chemosensory-like cells (thymic CCC) to designate this entity. Ultrastructure and cytokeratin content identify thymic CCC as members of the thymic epithelium. Although located in the medulla, they do not contain typical medullary cytokeratins, i.e., CK5 and CK14, but rather CK8 and CK18, which are characteristic for the majority of cortical epithelial cells (Shezen et al. 1995; Klug et al. 1998; Liepinsh et al. 2009; Lee et al. 2011). Notably, CK18 is also considered as a marker for oropharyngeal taste cells and various chemosensory surface epithelia (Kasper et al. 1993; Knapp et al. 1995; Höfer and Drenckhahn 1996; Höfer et al. 2000; Akimori et al. 2011). CK8 and CK18 form heterodimers and are the first intermediate filament proteins expressed during mouse embryogenesis in simple epithelia (Baribault et al. 1993). In the murine thymic medulla, a minor epithelial cell population expressing the cytokeratin pattern CK5^-^CK14^-^CK8^+^CK18^+^ has previously been noted, and these cells have been designated “globular” to distinguish them from the prevalent “stellate” CK5^+^CK14^+^ medullary epithelial cells (Klug et al. 1998; Lee et al. 2011). Their functional relevance has not been elucidated as yet.

A recent screen for taste receptor expression in nongustatory tissues by the genetic labeling of Tas2R131, a member of the bitter receptor family, has revealed single-positive cells in the murine thymic medulla, but these have not been characterized further (Voigt et al. 2012). A tempting speculation is that these cells at least partly overlap with the presently identified population, but Tas2R family members can also be expressed in non-chemosensory cells coupled to pathways distinct from those in taste cells (Grassin-Delyle et al. 2013; Lee et al. 2014). Tas2R131 in particular is also expressed in entirely unrelated cell types, such as sperm (Voigt et al. 2012), and in mouse colon in a subset of goblet cells, but explicitly not in chemosensory brush cells (Prandi et al. 2013).

In oropharyngeal taste buds, ATP is the major transmitter that excites sensory nerve fibers after tastant stimulation, and several other transmitters, among them ACh, are locally produced by sensory cells and also contribute to intragemmal signaling and modulation (Roper 2013). In chemosensory cells of the upper airways and trachea, ACh appears to be a major transmitter, and these chemosensory cells strongly express ChAT-eGFP in the mouse strains that have been used in the present study (Ogura et al. 2010; Krasteva et al. 2011, 2012; Saunders et al. 2014). Although we have noted a large overlap of ChAT-eGFP expression and immunoreactivity for PLCβ2 and TRPM5, these labels do not fully match 1:1, and taste transduction proteins (PLCβ2, TRPM5) have also been seen in a few ChAT-eGFP-negative cells. This resembles the situation in the trachea in which about 15 % of chemosensory cells do not express ChAT-eGFP (Krasteva et al. 2011). Whether this is attributable to the incomplete expression of the BAC transgene in cholinergic cells, as minor mismatches between transgene expression and ChAT immunolabeling also occur in the brain of these animals (Tallini et al. 2006; Engelhardt et al. 2007), or to the nonsynchronous expression of all markers at certain developmental stages or whether it reflects the existence of subpopulations (cholinergic and non-cholinergic) of thymic chemosensory-like cells remains to be established.

In contrast to chemosensory cells of the airways, which are connected to sensory nerve fibers and evoke reflex responses upon stimulation (Finger et al. 2003; Tizzano et al. 2010; Krasteva et al. 2011), we have not observed innervation of thymic CCC. Hence, a paracrine mode of cholinergic signaling within the thymus can be expected. Multiple muscarinic and nicotinic receptors are expressed by thymocytes and by myoid and thymic epithelial cells (Engel et al. 1977; Maśliński et al. 1987; Wakkach et al. 1996; Mihovilovic et al. 1997; Kuo et al. 2002; Poëa-Guyon et al. 2005), and effects elicited by cholinergic agonists are, accordingly, multifold. They include decreased cell adherence and growth of cultured thymic epithelial cells (Mihovilovic and Butterworth-Robinette 2001), increased release of lymphocytes into the circulation (Maśliński et al. 1987; Antonica et al. 1994), and arrest of thymocyte maturation at the double-positive stage in murine fetal thymus organ culture (Middlebrook et al. 2002). Among nAChR subunits, α3, α5 and β4, which can assemble into functional heteromers, exhibit the highest expression in early postnatal mouse thymus (Kuo et al. 2002), and we have noticed the expression of the ligand-binding subunit α3 in epithelial cells in direct contact to thymic CCC. We have recently characterized these nAChRα3-expressing cells as CK10-positive terminally differentiated epithelial cells of murine Hassall’s corpuscle-like structures (Soultanova et al. 2014), consistent with the occurrence in clusters also observed in the present study. The exact function of these cells is still unclear, but the spatial arrangement next to thymic CCC strongly suggests that they are under the control of cholinergic medullary paracrine signaling.

Epithelial chemosensory cells with the expression repertoire reported here for thymic CCC utilize canonical taste receptors to detect potential hazardous compounds at mucosal surfaces. In particular, they respond to bitter tasting bacterial products, including quorum-sensing molecules from *Pseudomonas aeruginosa*, so that they are considered as sentinels initiating protective reactions and reflexes to combat further ingression of bacteria or other harmful compounds (Tizzano et al. 2010; Ogura et al. 2010; Krasteva et al. 2011; Krasteva and Kummer 2012; Lee et al. 2014). In contrast to mucosal surfaces, the thymic medulla is not constantly exposed to inhaled or ingested foreign substances and bacteria. Nonetheless, it is reached by viruses, live bacteria, and bacterial products in systemic infection, and it harbors an infection-sensing pathway, whereby thymic epithelial cells drive programmed thymic involution (Dooley and Liston 2012). Whereas recent research has shed more light on the mechanism inducing rapid thymic involution in response to poly(I:C), which is structurally similar to viral double-stranded RNA, details of bacterial infection-sensing in the thymus are still largely unclear (Anz et al. 2009; Papadopoulou et al. 2011; Dooley and Liston 2012; Ross et al. 2012). The participation of thymic CCC in such intrathymic sensing mechanism appears to be an attractive possibility that should be further experimentally explored.
